# Diagnostic and Therapeutic Yield of Routine Esophagogastroduodenoscopy in Patients with Fontan Palliation

**DOI:** 10.1007/s00246-025-03988-1

**Published:** 2025-08-18

**Authors:** Josue Diaz-Frias, Hari S. Conjeevaram, Mark D. Norris, Michael R. Joynt, Sunkyung Yu, Ashley Duimstra, Timothy B. Cotts

**Affiliations:** 1 Division of Cardiovascular Medicine, Department of Internal Medicine, University of Michigan Health System, Ann Arbor, MI USA; 2 Division of Pediatric Cardiology, Department of Pediatrics, University of Michigan Health System, Ann Arbor, MI USA; 3 Division of Gastroenterology and Hepatology, Department of Internal Medicine, University of Michigan Health System, Ann Arbor, MI USA

**Keywords:** Congenital heart disease, Esophageal varices, Esophagogastroduodenoscopy, Extracardiac conduit, Fontan-associated liver disease, Fontan procedure

## Abstract

Fontan-associated liver disease (FALD) is an impactful complication for adults with Fontan circulation. The presence of esophageal varices is associated with increased morbidity and mortality in patients with underlying cirrhosis. The diagnostic yield of routine esophagogastroduodenoscopy (EGD) screening in Fontan patients with clinical or radiographic evidence of cirrhosis is not well known with wide practice variation across centers. We performed a retrospective chart review of all patients with Fontan circulation who underwent an EGD from 2020 to 2023 at our center. During the study period, a total of 66 patients (51.5% male) with Fontan circulation underwent a screening EGD at a median age 30.2 years (range 19.8–60.4 years). Esophageal varices were present in 41 (62.1%) patients, of which 35 (53.0%) had grade I esophageal varices, and 6 (9.1%) had grade II. The study showed a high incidence of esophageal varices in our patients with a Fontan circulation, suggesting a role for routine EGD as a screening tool in this population. This diagnostic procedure was well tolerated with no associated complications.

## Introduction

Over time, patients with Fontan Circulation develop a variety of long-term multi-organ pathology including the wide spectrum of Fontan-associated liver disease (FALD). These pathophysiological changes of the liver are thought to be caused by congestive hepatopathy secondary to major but not exclusive factors including chronically elevated central venous pressures and lack of pulsatility [1, 2]. While liver injury in Fontan patients is ubiquitous, it is unclear what proportion of patients and at what rate will develop clinically relevant advanced liver disease. Additionally, the presence of esophageal varices and other signs of portal hypertension have been associated with an increased mortality and morbidity risk in this population [3]. Many centers that care for adults with congenital heart disease have opted for the utilization of non-invasive diagnostic methods, with few reports of routine screening with esophagogastroduodenoscopy (EGD) [4].

A challenge in the management of FALD lies in the limited body of research and the absence of consensus-based, standardized surveillance guidelines. This gap in evidence contributes to considerable heterogeneity in clinical practice across institutions and highlights a significant unmet need in the longitudinal care of this complex patient population. Without uniform protocols, clinicians may rely on varied thresholds for evaluation and intervention, potentially delaying diagnosis or missing early markers of disease progression. Given this uncertainty, there is a pressing need for more studies that evaluate the diagnostic yield, risk–benefit balance, and long-term outcomes associated with different surveillance modalities in FALD. Such data would be instrumental in informing evidence-based guidelines and optimizing resource allocation in this growing adult congenital heart disease population. At our institution, the current practice involves referral to the hepatology for patients with clinical or radiographic features suggestive of FALD. If cirrhosis is suspected, based on imaging, laboratory data or clinical signs, patients who are clinically stable and have no contraindications undergo an EGD for further evaluation. This approach reflects a tailored, multidisciplinary decision-making process aimed at early identification of complications such as portal hypertension and esophageal varices. The primary aim of the study was to assess the diagnostic yield and safety of EGD in Fontan patients referred for FALD evaluation, with the goal of contributing to the development of a more strategic and evidence-based surveillance framework. By evaluating procedural outcomes and clinical correlates, we sought to clarify the potential role of EGD in routine surveillance and risk stratification in this unique and high-risk population.

## Materials and Methods

### Study Population and Design

This was an exploratory single-center retrospective chart review of de-identified data of all adult patients (age > 18 years) with a Fontan circulation who underwent a screening EGD from February 1, 2020, to October 31, 2023, at University of Michigan Health System. The study was approved by the Institutional Review Board.

### Study Objectives

Our primary objective was to assess the overall diagnostic and therapeutic yield of EGD in the adult Fontan population, specifically in the diagnosis of esophageal varices and associated conditions. Secondary objectives included determining procedural safety and risks, and incidence of therapeutic interventions.

### Descriptive Data

Variables collected included age, sex, race/ethnicity, height, weight, body mass index (BMI), underlying cardiac anatomy, age at stage 2 palliation, age at Fontan completion, type of Fontan, the presence of heterotaxy syndrome, the presence of a fenestration, oxygen saturation at rest, systolic and diastolic blood pressure, supplemental oxygen at rest, pulmonary and systemic antihypertensive therapy, the presence of a permanent pacemaker, history of recurrent arrhythmias, New York Heart Association (NYHA) functional class, and the presence of protein losing enteropathy or plastic bronchitis. Esophageal varices were graded using a size-based classification system (F1, F2, F3), with varices categorized as small if < 5 mm in diameter and large if ≥ 5 mm, consistent with the Japanese Research Society of Portal Hypertension (JRSPH). Gastric varices, when present, were characterized using the Sarin classification, which categorizes varices by their anatomical location.

Laboratory studies and other non-invasive or invasive diagnostic studies and tests done within one-year pre- and three months post-EGD procedure were reviewed. These included echocardiograms, cardiac magnetic resonance imaging (MRI), cardiac catheterization, abdominal ultrasound (US), chest computed tomography (CT) or MRI, elastography, liver biopsies, cardiopulmonary exercise stress test (CPET) results, liver function tests, brain natriuretic peptide (BNP), gamma-glutamyl transferase (GGT), alpha-fetoprotein (AFP), platelets, and INR.

### Data Analysis

Data are reported as frequency with percentage (%) for categorical variables and median (interquartile range), mean ± standard, or a full range (minimum to maximum) for continuous variables, as appropriate. A cumulative incidence of the presence of esophageal varices during the study period was calculated and reported. Univariate associations of demographics and clinical characteristics with the presence of esophageal varices were examined using Chi-square test or Fisher’s exact test for categorical variables and Wilcoxon rank sum test for continuous variables. All analyses were performed with SAS Version 9.4 (SAS institute Inc., Cary, NC). A *P* value < 0.05 was considered statistically significant.

## Results

During the study period, a total of 66 adult patients with Fontan circulation underwent routine EGD at a median age of 30.2 years (range 19.8–60.4 years) and a median time from Fontan palliation of 27.1 years (ranging from 12.3 to 45.1 years). Patient demographics and clinical characteristics are shown in Table 1. Over half of the patients (53.0%) were NYHA functional class II, suggesting a predominance of moderate functional limitation, and nearly one-third (28.8%) was classified as class III, indicating a substantial burden of symptoms in a notable portion of the cohort. The Fontan type was lateral tunnel in 41 patients (62.1%), atrio-pulmonary in 14 (21.2%), and extracardiac in 11 (16.7%). Regarding underlying cardiac diagnoses, hypoplastic left heart syndrome (HLHS) was most common (37.9%), followed by double inlet left ventricle (21.2%), tricuspid atresia (15.2%), and unbalanced atrioventricular septal defect (AVSD) (10.6%). In addition, systemic ventricular morphology was relatively evenly distributed, with a slight predominance of systemic right ventricles observed in 34 patients (51.5%).

**Table 1:** Demographics and medical history/comorbidities (*N* = 66)

Characteristic	All (*N* = 66)	Esophageal varices present		*P* value^§^
		No (*N* = 25)	Yes (*N* = 41)	
Demographics				
Male sex	34 (51.5)	8 (32.0)	26 (63.4)	0.01
Caucasian race	61 (92.4)	23 (92.0)	38 (92.7)	1.00
Hispanic ethnicity	3 (4.6)	2 (8.0)	1 (2.4)	0.55
Cardiac anatomy and history				
Systemic ventricle				0.57
Left	32 (48.5)	11 (44.0)	21 (51.2)	
Right	34 (51.5)	14 (56.0)	20 (48.8)	
Underlying cardiac diagnosis				0.19^†^
HLHS	25 (37.9)	12 (48.0)	13 (31.7)	
DILV	14 (21.2)	3 (12.0)	11 (26.8)	
DORV	4 (6.1)	1 (4.0)	3 (7.3)	
PA	2 (3.0)	0 (0.0)	2 (4.9)	
PA/IVS	2 (3.0)	1 (4.0)	1 (2.4)	
Tricuspid atresia	10 (15.2)	4 (16.0)	6 (14.6)	
Unbalanced AV canal	7 (10.6)	3 (12.0)	4 (9.8)	
Other	2 (3.0)	1 (4.0)	1 (2.4)	
Stage 2 palliation^a^	45 (68.2)	18 (72.0)	27 (65.9)	0.60
Age at stage 2 palliation, months	6.9 (6.0–11.2)	6.9 (5.9–11.0)	7.0 (6.1–11.5)	0.87
Age at Fontan palliation, years	2.7 (1.9–5.1)	2.6 (2.0–5.4)	2.8 (1.9–4.5)	0.68
Type of Fontan				0.34
Lateral tunnel	41 (62.1)	17 (68.0)	24 (58.5)	
Atrio-pulmonary	14 (21.2)	6 (24.0)	8 (19.5)	
Extracardiac conduit	11 (16.7)	2 (8.0)	9 (22.0)	
Fenestration created	41 (62.1)	17 (68.0)	24 (58.5)	0.44
Functional status				
NYHA classification at EGD				0.35
I	12 (18.2)	3 (12.0)	9 (22.0)	
II	35 (53.0)	16 (64.0)	19 (46.3)	
III	19 (28.8)	6 (24.0)	13 (31.7)	
Fontan-associated and cardiac-related factors				
Permanent pacemaker	15 (22.7)	3 (12.0)	12 (29.3)	0.10
History of recurrent arrhythmias	35 (53.0)	11 (44.0)	24 (58.5)	0.25
Heterotaxy present	6 (9.1)	3 (12.0)	3 (7.3)	0.67
Plastic bronchitis	1 (1.5)	0 (0.0)	1 (2.4)	1.00
Protein-losing enteropathy	5 (7.6)	2 (8.0)	3 (7.3)	1.00
Hepatic factors				
Hepatitis C	2 (3.0)	0 (0.0)	2 (4.9)	0.52
History of hepatocellular carcinoma	2 (3.0)	1 (4.0)	1 (2.4)	1.00
Biliary atresia s/*p* Kasai	1 (1.5)	0 (0.0)	1 (2.4)	1.00
Pulmonary Factors				
History of pulmonary embolism	6 (9.1)	4 (16.0)	2 (4.9)	0.19
Supplemental oxygen required at rest	6 (9.1)	3 (12.0)	3 (7.3)	0.67
Pulmonary hypertensive therapy required	12 (18.2)	4 (16.0)	8 (19.5)	1.00
Other comorbidities/factors				
Obesity	26 (39.4)	13 (52.0)	13 (31.7)	0.10
Chronic kidney disease/end-stage renal disease	5 (8.6)	0 (0.0)	5 (12.2)	0.15
History of atrial/IVC/Fontan pathway thrombosis	6 (9.1)	1 (4.0)	5 (12.2)	0.40
Systemic hypertensive therapy	37 (56.1)	13 (52.0)	24 (58.5)	0.60
Chronic venous insufficiency	7 (10.6)	2 (8.0)	5 (12.2)	0.70
Obstructive sleep apnea	15 (22.7)	6 (24.0)	9 (22.0)	0.85
History of deep vein thrombosis	3 (4.6)	1 (4.0)	2 (4.9)	1.00
History of GI bleeding	1 (1.5)	0 (0.0)	1 (2.4)	1.00

*Data are presented as N (%) for categorical variables and Median (interquartile range) for continuous variables

^§^*P* value from Chi-square test or Fisher’s exact test for categorical variables and Wilcoxon rank sum test for continuous variables

^†^Comparison was made as HLHS vs. all other primary cardiac diagnoses and *P* value came from Chi-square test

^a^*N* = 44 for stage 2 palliation age due to incomplete early surgical records

Notably, only one patient (1.5%) had a prior history of grade II esophageal varices with gastrointestinal bleeding requiring endoscopic banding (Table 1). Recurrent atrial arrhythmias were present in over half of the patients (53.0%). Obesity was present in 39.4% of patients, and other comorbidities include obstructive sleep apnea (22.7%), chronic venous insufficiency (10.6%), history of Fontan pathway thrombosis, pulmonary embolism (9.1%), and chronic kidney disease (7.6%). Importantly, two patients had a history of hepatocellular carcinoma (HCC), and 15 (22.7%) patients had a permanent pacemaker.

Esophageal varices were identified in a substantial proportion of the cohort (62.1%), with the majority (*n* = 35, 53.0%) presenting with grade I varices, and a smaller subset (*n* = 6, 9.1%) exhibiting grade II varices (Table 2). Notably, no advanced varices (grade III or IV) were observed. Additional endoscopic findings included portal hypertensive gastropathy in half of the cohort (50.0%), erythematous mucosal changes in 16 patients (24.2%), non-bleeding erosive gastropathy in 4 patients (6.1%), and gastric vascular ectasia in 1 patient (1.5%). The presence of esophageal varices was significantly more common in male patients (*p* = 0.01), suggesting potential sex-based difference in the pathophysiology or progression of FALD. Additionally, a trend toward increased incidence of varices was noted among patients with permanent pacemaker implanted (*p* = 0.10; Table 1). In the non-varices group, all three pacemaker recipients had sinus node dysfunction as the underlying indication. In contrast, among those with varices, 66.7% (*n* = 8) had sinus node dysfunction, 16.7% (*n* = 2) had sinus node dysfunction but were predominantly ventricular paced, and 16.7% (*n* = 2) had complete heart block.

**Table 2:** EGD findings (*N* = 66)

Age at EGD, years	30.2 (26.5–38.7)
Time from Fontan to EGD, years	27.1 (23.6–33.4)
Height at EGD, cm	168 ± 9.6
Weight at EGD, kg	78.9 ± 19.6
BMI at EGD	28.0 ± 6.5
SpO_2_ at EGD, %	91.3 ± 5.3
Systolic BP at EGD, mmHg	120 ± 19.5
Diastolic BP at EGD, mmHg	66.3 ± 10.6
Any abnormal EGD finding	55 (83.3)
Esophageal varices	41 (62.1)
Portal hypertensive gastropathy	33 (50.0)
Erythematous mucosa in the antrum	11 (16.7)
Non-bleeding erosive gastropathy	4 (6.1)
Erythematous mucosa in the gastric body	3 (4.6)
Erythematous mucosa in the entire examined stomach	2 (3.0)
Duodenopathy	3 (4.6)
Esophagitis (Grade A)^a^	2 (3.0)
Other findings^b^	5 (7.6)
Therapeutic intervention during EGD	0 (0.0)

*Data are presented as *N* (%) for categorical variables and median (interquartile range) or mean ± standard deviation for continuous variables

^a^Esophagitis graded using Los Angeles Classification grading system

^b^Other findings include diffuse mucosal friability (*n* = 1), duodenal erythematous mucosa (*n* = 1), gastric antral vascular ectasia (*n* = 1), hiatal hernia (*n* = 1), and type 2 gastroesophageal varices (*n* = 1)

All 66 patients in the cohort underwent transthoracic echocardiography within the study period, while a subset underwent more advanced hemodynamic and functional assessment, including cardiac MRI in 17 patients (25.8%), cardiac catheterization in 14 patients (21.2%), and CPET in 26 patients (39.4%) (Table 3). Echocardiographic assessment revealed that just over half of the patients (51.5%) had a normal or low-normal systemic ventricular systolic function, while moderately or greater than moderately depressed systolic function was observed in 15.2% of the patients. Valvular disease was relatively limited in severity. Most patients had none or mild atrioventricular (AV) valve regurgitation (84.9%), with moderate or greater regurgitation observed in 6.1%. No patient had aortic insufficiency greater than mild, and a patent fenestration was visualized in 18.2% of patients. There were no differences in these echocardiographic features between patients with and without esophageal varices (Table 5). Among the 14 patients who underwent cardiac catheterization, the mean Fontan pathway pressure was 17.4 mmHg, and the average capillary wedge pressure was 12.5 mmHg. In the routine baseline laboratory surveillance (Table 4), elevated total bilirubin (> 1.2 mg/dL) was observed in 26 (39.4%) patients, suggesting early cholestasis or impaired hepatic clearance. Liver enzymes were elevated in a smaller proportion of patients, with AST > 40 U/L in 6 (9.1%) patients, ALT > 40 U/L in 8 (12.3%) patients, and alkaline phosphatase (ALP) > 150 IU/L in 6 (9.1%) patients. Thrombocytopenia (platelets < 150,000/μL), a common surrogate marker of portal hypertension and hypersplenism in FALD, was present in nearly half of the cohort (*n* = 31, 47.0%). Additionally, 11/44 (25.0%) patients with available data of B-type natriuretic peptide (BNP) had BNP > 100 pg/mL.

**Table 3:** Cardiac diagnostic studies (*N* = 66)

Echocardiogram	
Systolic function^a^	
Normal/low normal	34 (51.5)
Mildly depressed	17 (25.8)
Mild-moderately depressed	5 (7.6)
Moderately depressed	7 (10.6)
Moderate-severely depressed	3 (4.6)
AV valve regurgitation	
None	23 (34.9)
Mild	33 (50.0)
Mild-moderate	6 (9.1)
Moderate	3 (4.6)
Moderate-severe	1 (1.5)
Semilunar valve regurgitation	
None	47 (71.2)
Mild	19 (28.8)
Fenestration present^b^	12 (18.2)
Fontan pathway patent	66 (100.0)

Cardiac MRI	*n* = 17 (25.8%)
Systolic function	
Normal/low normal	8 (47.1)
Mildly depressed	7 (41.2)
Mild-moderately depressed	1 (5.9)
Moderately depressed	1 (5.9)
AV valve regurgitation	
None	2 (11.8)
Mild	10 (58.8)
Mild-moderate	1 (5.9)
Moderate	2 (11.8)
Severe	1 (5.9)
Unknown	1 (5.9)
Semilunar valve regurgitation	
None	14 (82.4)
Moderate	2 (11.8)
Unknown	1 (5.9)
Fenestration present^b^	2 (11.8)
Fontan pathway patent	17 (100.0)

Cardiac catheterization^c^		Range (Min–Max)
Fontan pathway pressure, mmHg	17.4 ± 3.2	11–22
Capillary wedge pressure, mmHg	12.5 ± 3.0	8–19
Fenestration present^b^	2 (14.3)	

Cardiopulmonary exercise stress test^d^			
	Baseline/resting	Peak	Post (3 min. recovery)
Heart rate, bpm	76.0 ± 16.9	140 ± 33.5	102 ± 20.7
Systolic BP, mmHg	112 ± 11.1	148 ± 19.8	124 ± 17.5
Diastolic BP, mmHg	66.4 ± 9.6	62.9 ± 7.9	64.2 ± 9.0
SpO_2_, %	91.6 ± 5.1	86.2 ± 7.3	89.8 ± 3.7
VO_2_	4.4 ± 1.5	20.1 ± 7.1	N/A

^a^Systolic function was assessed qualitatively by visual estimation

^b^Fenestration refers to an anatomical fenestration, identified through imaging studies (Echocardiogram or cardiac MRI) or cardiac catheterization

^c^*N* = 14 patients underwent a cardiac catheterization

^d^*N* = 26 patients underwent a cardiopulmonary exercise stress test

*Data are presented as N (%) for categorical variables and mean ± standard deviation and range (min-max) for continuous variables

**Table 4:** Surveillance laboratory results (N = 66)

Laboratory parameter	Data*	Range	References
^‡^AFP, ng/mL	2.4 (< 2.0–4.0)	1.7–1,722	< 10
ALP, U/L	95.3 ± 34.9	31.0–205	44–147
ALT, U/L^a^	30.1 ± 21.4	< 7.0–164	7–56
AST, U/L	29.3 ± 14.3	15.0–126.0	10–40
^†^BNP, pg/mL^b^	59.5 (27.5–106)	6.0–468	< 100^†^
GGT, U/L^c^	64.0 (41.0–137)	27–342	9–48 (♂)
			5–36 (♀)
INR^d^	1.2 (1.1–1.6)	1.0–3.1	0.8–1.2
Platelets, k/uL	159 ± 52.4	59.0–286	150–400
Total bilirubin, mg/dL	1.1 (0.7–1.5)	0.3–4.3	0.1–1.2

^†^BNP values may vary with age, sex, and volume status, they were not age- or sex-adjusted

^‡^AFP values were not age- or sex-adjusted. In this cohort, two patients who had AFP > 10 ng/mL had hepatocellular carcinoma (HCC)

^a^*N* = 65 patients had an ALT

^b^*N* = 44 patients had a BNP

^c^*N* = 25 patients had a GGT

^d^*N* = 61 patients had an INR

*Data are presented as mean ± standard deviation or median (interquartile range) and range [min–max]

A univariate comparison between Fontan patients with and without esophageal varices showed several notable differences in laboratory hepatic parameters (Table 5). Mean AST was significantly increased among patients with varices compared to those without varices (31.9 ± 16.7 vs. 25.0 ± 7.4 U/L, *p* = 0.03). Similarly, mean ALT was significantly higher in patients with varices (33.8 ± 25.7 U/L) compared to those without varices (24.2 ± 9.0 U/L) (*p* = 0.04). These elevations suggest a greater degree of hepatocellular injury or stress in patients with established portal hypertension manifesting as varices. Although not statistically significant, platelet count, an established surrogate marker of portal hypertension, was lower in patients with varices (150 ± 54.0 vs. 175 ± 46.6 K/μL, *p* = 0.06). This trend may reflect hypersplenism or splenic sequestration secondary to portal hypertension. Non-invasive fibrosis scoring systems such as the AST-to-platelet ratio index (APRI) and the Fibrosis-4 index (FIB-4) were also calculated. Among patients without esophageal varices, a low APRI score (< 0.5) was observed in 88.0%, whereas only 43.9% of patients with varices fell within this range (*p* = 0.0004), suggesting statistically significant inverse association between lower APRI values and the presence of esophageal varices. Conversely, a high APRI score (> 1.5) was rare, occurring in only 2.4% of those with varices (*p* = 1.00), reflecting a low prevalence of markedly elevated APRI in the cohort and limiting its discriminative utility for varices at this threshold. The FIB-4 index showed a non-significant trend toward association with varices. A low FIB-4 score (< 1.45) was observed on 84.0% of patients without varices and 67.5% of those with varices (*p* = 0.14), suggesting a potential, but not statistically significant, protective trend associated with lower FIB-4 values. Similarly, a high FIB-4 score (> 3.25) was absent in patients without varices but present in 10.0% of those with varices (*p* = 0.15). While these results do not reach statistical significance, the directionality is consistent with FIB-4 reflecting increasing fibrosis and potential portal hypertension.

**Table 5:** Univariate comparisons of laboratory and echocardiographic characteristics with presence of esophageal varices on EGD (*N* = 66)

Characteristic	No varices present (*N* = 25)	Varices present (*N* = 41)	*P* value^§^
Labs			
AFP, ng/mL	3.0 (< 2.0–4.0)	< 2.0 (< 2.0–4.0)	0.63
ALP, U/L	89.7 ± 30.6	98.7 ± 37.2	0.31
ALT, U/L^a^	24.2 ± 9.0	33.8 ± 25.7	**0.04**
AST, U/L	25.0 ± 7.4	31.9 ± 16.7	**0.03**
BNP, pg/mL^b^	61.0 (16.0–92.0)	59.0 (30.0–113)	0.81
GGT, U/L^c^	44.5 (35.0–110)	97.0 (49.0–147)	0.18
INR^d^	1.2 (1.1–1.6)	1.3 (1.1–1.7)	0.47
Platelets, k/µL	175 ± 46.6	150 ± 54.0	**0.06**
Total bilirubin, mg/dL	0.90 (0.70–1.3)	1.1 (0.80–1.7)	0.27
Non-invasive fibrosis scoring s			
APRI	0.34 (0.30–0.46)	0.57 (0.38–0.73)	**0.002**
Low APRI (< 0.5)	22 (88.0)	18 (43.9)	**0.0004**
High APRI (> 1.5)	0 (0.0)	1 (2.4)	1.00
FIB-4^e^	0.83 (0.70–1.2)	1.2 (0.81–1.7)	0.12
Low FIB-4 (< 1.45)	21 (84.0)	27/40 (67.5)	0.14
High FIB-4 (> 3.25)	0 (0.0)	4/40 (10.0)	0.15
Echocardiogram			
Systolic function			1.00
< Moderately depressed	21 (84.0)	35 (85.4)	
≥ Moderately depressed	4 (16.0)	6 (14.6)	
AV valve regurgitation			0.63
< Moderately depressed	23 (92.0)	39 (95.1)	
≥ Moderately depressed	2 (8.0)	2 (4.9)	
Semilunar valve regurgitation			N/A
< Moderately depressed	25 (100.0)	41 (100.0)	
≥ Moderately depressed	0 (0.0)	0 (0.0)	
Fenestration present	4 (16.0)	8 (19.5)	1.00

*Data are presented as *N* (%) for categorical variables and mean ± standard deviation or median (interquartile range) for continuous variables

^§^P value from Fisher’s exact test for categorical variables and two-sample t test or Wilcoxon rank sum test for continuous variables

^a^*N* = 65 patients had an ALT

^b^*N* = 44 patients had a BNP

^c^*N* = 25 patients had a GGT

^d^*N* = 61 patients had an INR

^e^On *N* = 65 patients a FIB-4 score was obtained, as only 65 patients had an ALT value reported

Majority of patients (*n* = 55, 83.3%) underwent an abdominal US within the study period. Among these, 52 patients (94.6%) demonstrated nodular hepatic morphology, a hallmark of hepatic architectural remodeling. Splenomegaly, a surrogate marker of portal hypertension, was observed in 61.8% of the patients, while 32.7% had radiographic evidence of ascites and 29.1% exhibited hepatic masses (Table 6). Abdominal MRI was performed in 40 (60.6%) patients, with 97.5% showing nodular liver changes. Elevated liver stiffness, suggestive of advance fibrosis, was present in 55.0% of the patients, and 62.5% had splenomegaly. Additionally, ascites and liver nodules were identified in 25.0% and 45.0%, respectively, while esophageal varices were visualized in 22.5% (*n* = 9) of the patients. For those 9 patients with esophageal varices observed by an abnormal MRI, 5 patients were identified by EGD, resulting in sensitivity of 25.0%, specificity of 80.0%, positive predictive value of 55.6%, and negative predictive value of 51.6% for detecting varices by MRI, which highlight the limitations of MRI for direct variceal detection. Only 19 patients (28.8%) had an abdominal CT, with all of them demonstrating nodular changes of the liver, 9 patients (47.4%) had ascites, 8 patients (42.1%) had splenomegaly, 7 patients (36.8%) had liver nodules, and 5 patients (26.3%) had esophageal varices. Like MRI, an abdominal CT showed poor diagnostic performance to detecting varices; of those 5 patients with esophageal varices observed by an abnormal CT, 3 patients were identified by EGD—sensitivity of 21.4%, specificity of 60.0%, positive predictive value of 60.0%, and negative predictive value of 21.4%. Liver elastography was performed in 34 patients (51.5%), with 17.6% of the patients demonstrating elevated stiffness values (> 7 kPa), indicative of significant fibrosis. Elastography was conducted via MRI in 22 patients and by US/FibroScan in 12 patients, emphasizing the utility of multimodal, non-invasive techniques in evaluating hepatic involvement in the Fontan population.

**Table 6:** Hepatic diagnostic studies among Fontan patients (*N* = 66)

Abdominal ultrasound	*N* = 55 (83.3%)
Any abnormal finding	54 (98.2)
Nodular changes to liver	52 (94.6)
Splenomegaly	34 (61.8)
Ascites	18 (32.7)
Nodules	16 (29.1)
Liver stiffness^‡^	1 (1.8)
Liver enlargement	1 (1.8)
Other^a^	7 (12.7)

Abdominal MRI	*N* = 40 (60.6%)
Any abnormal finding	40 (100.0)
Nodular changes to liver	39 (97.5)
Splenomegaly	25 (62.5)
Liver stiffness^‡^	22 (55.0)
Nodules	18 (45.0)
Ascites	10 (25.0)
Liver enlargement	8 (20.0)
Other^b^	12 (30.0)

Abdominal CT	*N* = 19 (28.8%)
Any abnormal finding	19 (100.0)
Nodular changes to liver	19 (100.0)
Ascites	9 (47.4)
Splenomegaly	8 (42.1)
Nodules	7 (36.8)
Liver enlargement	3 (15.8)
Other^c^	6 (31.6)

Elastography	*N* = 34 (51.5%)
Stiffness value, kPa	5.0 (3.9–6.1)
Quantitative fat, % (*N* = 22)	3.5 (2.8–4.5)
Findings	13 (38.2)
Fatty liver^†^	10 (29.4)
Other^d^	3 (8.8)

Liver biopsy	*N* = 3 (4.6%)
Degree of fibrosis	
3	1 (33.3)
4	2 (66.7)

^a^Other US findings include 6 cholelithiasis (*n* = 6) and fatty liver (*n* = 1)

^b^Other MRI findings include fatty liver (*n* = 3), abdominal varices (*n* = 2), upper abdominal varices (*n* = 2), gastroesophageal varices (*n* = 1), cholelithiasis and gastric, mesenteric and paraesophageal varices (*n* = 1), cholelithiasis and upper abdominal varices (*n* = 1), fatty liver and mesenteric, upper abdominal and abdominal wall varices (*n* = 1), upper abdominal and gastroesophageal varices (*n* = 1)

^c^Other CT findings include varices (esophageal, paraoesophageal, abdominal, epigastric) (*n* = 5), and possible fatty infiltration (*n* = 1)

^d^Other Elastography findings include cholelithiasis (*n* = 1), nodular changes (*n* = 1), and splenomegaly (*n* = 1)

^‡^Liver stiffness by US was radiologist-defined (not quantitative). Liver stiffness by MRI/MRE was defined with a cutoff ≥ 3.0 kPa (indicative of possible fibrosis)

^†^Fatty liver was based on increased echogenicity; radiologist-defined (not quantitative)

*Data are presented as *N* (%) for categorical variables and median (interquartile range) for continuous variables

## Discussion

With the growing population of adults surviving into adulthood following Fontan palliation, there is a rising clinical imperative to address long-term complications associated with Fontan physiology. Despite this increasing demand, surveillance practices for Fontan-associated complications remain inconsistent across institutions, reflecting the lack of standardized, evidence-based guidelines. This variability poses challenges in timely detection and management of end-organ sequelae and contributes to disparities in care delivery. FALD is now recognized as an almost universal consequence of the Fontan circulation, resulting from chronically elevated systemic venous pressure and diminished hepatic perfusion. Over time, this unique hemodynamic state promotes hepatic fibrosis, architectural remodeling, and eventually cirrhosis. As FALD advances, patients are at increased risk of developing portal hypertension and its sequelae, including ascites, splenomegaly, and esophageal varices. Moreover, the substantial multisystem burden resulting from the numerous comorbidities commonly seen in the adults with Fontan physiology may further compromise systemic hemodynamics and contribute to the accelerated progression of hepatic dysfunction. The presence of ventricular dysfunction, elevated Fontan pressures, biochemical markers of hepatic congestion, and thrombocytopenia underlines the complex and progressive nature of late-stage Fontan physiology. These objective clinical parameters may serve as important adjuncts to risk stratification and ongoing surveillance, particularly when evaluating the need for diagnostic interventions [5, 6]. While the utility of non-invasive imaging modalities in directly identifying esophageal varices remains limited, these techniques nonetheless provide important complementary information in the evaluation of FALD. Cross-sectional imaging frequently demonstrates hepatic surface nodularity, parenchymal heterogeneity, and other morphological changes suggestive of hepatic fibrosis or the presence of regenerative nodules. However, a notable diagnostic limitation persists in reliably distinguishing these benign hepatic alterations from early HCC, particularly in the setting of chronic congestion and progressive liver remodeling. As such, while imaging plays a critical role in longitudinal surveillance, its findings must be interpreted within the broader clinical and hemodynamic context, ideally, supplemented by additional diagnostic modalities. Importantly, the risk of HCC increases with time post-Fontan, particularly in those with advanced fibrosis or cirrhosis, emphasizing the need for vigilant hepatic surveillance strategies [6]. These observations highlight a critical need for the development of standardized surveillance protocols tailored to the Fontan population, particularly regarding hepatic monitoring, risk stratification, and early identification of complications such as varices and HCC. Therefore, greater emphasis must be placed on evaluating the clinical utility and appropriateness of relatively invasive diagnostic modalities. Esophagogastroduodenoscopy (EGD), while traditionally used for diagnostic and therapeutic purposes in patients with established cirrhosis, may also hold value as a screening tool in selected subsets of the Fontan population, particularly those with clinical, laboratory, or imaging evidence suggestive of advanced liver disease. Our study supports this notion, demonstrating that EGD was not only well tolerated but also effective in identifying early-stage esophageal varices in a substantial proportion of patients. Importantly, there were no reported peri-procedural complications, underlining the safety profile of EGD in this complex population when performed under appropriate clinical circumstances. These findings suggest that, when guided by clinical or radiographic indicators of cirrhosis, EGD may serve as a valuable adjunct in risk stratification and proactive management of portal hypertension-related complications. However, further studies are needed to refine patient selection criteria, determine cost-effectiveness, and assess the long-term impact of EGD-guided surveillance on clinical outcomes in adults with a Fontan circulation.

All patients who underwent EGD in our cohort were clinically compensated at the time of the procedure, with no evidence of decompensated cirrhosis. Esophageal varices were identified in 62.1% of patients, substantially higher than the prevalence reported in prior endoscopic studies, including 9.3% by Agnoletti et al., 27% by Chang et al., and 34% by Ahmed et al. [1, 4, 7]. These also exceed those observed through non-invasive imaging modalities, which have demonstrated a range from 9.4 to 50% [1–3, 6]. The higher prevalence of esophageal varices in our study, relative to prior reports, may be attributable, at least in part, to the proactive approach employed at our institution, wherein EGDs were routinely performed by the hepatology service as part of a targeted screening protocol in patients with clinical or imaging evidence suggestive of cirrhosis.

Additionally, the presence of esophageal varices in Fontan patients carries significant clinical implications, particularly due to the potential of upper gastrointestinal bleeding, which can disrupt the already tenuous hemodynamic equilibrium inherent to Fontan physiology. Notably, the incidence of new esophageal varices in serial endoscopic surveillance has been reported to reach up to 9% annually, suggesting a progressive nature of portal hypertension in this population, as noted in a recent study by Ahmed et al. In this study, the annual incidence of gastrointestinal bleeding in patients with FALD and esophageal varices was 5%, with a 5-year cumulative incidence of 26% [7]. In our study, only one patient with a prior history of grade II esophageal varices and associated gastrointestinal bleeding underwent therapeutic band ligation prior to the study period. No cases of active variceal bleeding were identified during routine surveillance EGDs. All procedures were performed under moderate sedation with involvement of a dedicated congenital cardiac anesthesia team to ensure hemodynamic stability. Importantly, no patients required endoscopic intervention at the time of EGD, and no procedural complications were reported. Post-procedure management primarily involved outpatient hepatology follow-up, with most patients scheduled for a repeat EGD surveillance within 1 to 2 years, consistent with a conservative, risk-based monitoring approach. These findings highlight the high burden of subclinical gastrointestinal complications in the adult Fontan population and support the utility of EGD in detecting early portal hypertensive changes, which may have complications for long-term surveillance and intervention strategies.

Esophageal varices were observed more frequently among male patients in our cohort, with a trend toward higher incidence in patients with a permanent pacemaker, suggesting a possible association between chronic ventricular pacing and the development of portal hypertension in patients with Fontan physiology. Notably, in the non-varices group, all three patients with pacemakers had sinus node dysfunction, without reliance on ventricular pacing. In contrast, the varices group demonstrated more heterogeneous pacing indications, with approximately a third of patients relying on ventricular pacing. This distribution implies that the burden of ventricular pacing, especially in the absence of atrioventricular synchrony, may contribute to hemodynamic derangements that exacerbate FALD. Chronic ventricular pacing may impair diastolic filling, reduce effective pulmonary blood flow, and increase central venous pressure, all of which can accelerate hepatic congestion and fibrosis. While causality cannot be inferred from this small subgroup analysis, these findings warrant further investigation in larger, multicenter cohorts, investigating pacing modality, ventricular pacing burden, and timing of pacemaker implantation in the Fontan population. In addition, laboratory indicators of hepatic stress, particularly elevated transaminases and trend toward thrombocytopenia, were associated with the presence of esophageal varices. We also evaluated the association between non-invasive liver fibrosis scoring systems and the presence of esophageal varices. These indices such as APRI and FIB-4 are frequently utilized in assessing hepatic fibrosis severity and may serve as a surrogate marker for portal hypertension complications, including esophageal varices. Based on our results, a low APRI score demonstrated a significant inverse association with the presence of esophageal varices and may be useful in identifying patients at low risk. A high APRI and FIB-4 scores were uncommon in this cohort and did not show statistically significant associations with varices, likely due to low event rates and sample size. Lastly, a low FIB-4 trended toward being protective against varices, though not statistically significant. These findings support further investigation into the utility of non-invasive scoring systems in risk stratification for portal hypertension-related complications, particularly in unique populations such as those with Fontan physiology. It is important to also mention that in our study both abdominal MRI and CT modalities have limited diagnostic performances for detecting esophageal varices in Fontan patients, despite reasonable specificity. While these imaging modalities remain valuable for assessing liver morphology, collateral vasculature, and stiffness on MRI, our results suggest that it should not be relied upon as a sole diagnostic modality for ruling in or out varices. Therefore, cross-sectional imaging, while useful for global hepatic and vascular assessment, may not be able to replace EGD for variceal screening or surveillance in this population at the present time. Further studies could explore whether certain advanced cross-sectional techniques or dedicated variceal reporting protocols could improve diagnostic yield.

## Study Limitations

Due to the retrospective nature of our analysis, we are limited in our ability to determine causality or assess the potential influence of unmeasured confounding variables on the development of varices. The lack of blinding introduces the potential for assessment bias, and both selection and information biases may have influenced the observed findings. For instance, patients undergoing EGD may have been selectively referred based on clinical suspicion or institutional protocols, thereby limiting the generalizability of the results to broader Fontan populations. Furthermore, the study was restricted to descriptive and univariate analyses. The study cohort consisted predominantly of Caucasian patients and was drawn from a single quaternary care center with an institution-specific surveillance protocol, which may not reflect practices or patient demographics at other institutions. These factors collectively constrain the external validity of the findings. Future prospective, multicenter studies employing standardized screening algorithms are needed to validate these observations and to better inform clinical guidelines for the surveillance and management of FALD.

Nonetheless, our findings raise important considerations for clinical practice and underline the need for more rigorous prospective studies to elucidate risk factors and optimize surveillance strategies. In the absence of established consensus guidelines for the diagnosis and monitoring of FALD, there is a pressing need for collaborative research efforts that integrate the expertise of both cardiology and hepatology disciplines. Such multidisciplinary initiatives will be critical for the development of standardized protocols that can inform clinical decision-making and reduce practice variability. Based on the results of our study, EGD may be a valuable screening tool in Fontan patients with evidence of hepatic cirrhosis, either clinically or radiographically, as it allows for the early identification of esophageal varices, a potentially life-threatening complication. Importantly, all procedures were well tolerated and completed without peri-procedural complications, reflecting the benefit of care in specialized centers with expertise in patients with adult congenital heart disease, incorporating specialized anesthesia support and multidisciplinary risk stratification into routine practice.

## Conclusion

Esophagogastroduodenoscopy (EGD), when incorporated as part of a structured screening protocol in Fontan patients with clinical or radiographic evidence of Fontan-associated liver disease (FALD), appears to be a well-tolerated, low-risk procedure when performed with appropriate multidisciplinary risk mitigation strategies. In our cohort, EGD demonstrated a relatively high diagnostic yield for esophageal varices, a clinically significant complication with potential hemodynamic implications in this population. Given the absence of consensus guidelines and the limitations of non-invasive imaging in reliably detecting varices, our findings suggest that EGD may serve as a valuable adjunctive tool in the comprehensive surveillance of Fontan patients at risk for portal hypertension-related complications. However, further prospective, multicenter cohort studies are needed to directly compare EGD with emerging non-invasive diagnostic modalities, to clarify the natural history and progression of esophageal varices in this population, and to assess long-term clinical outcomes such as bleeding risk, need for endoscopic intervention, and overall survival impact. Development of evidence-based, multidisciplinary screening algorithms will be critical in the optimizing care for the growing adult Fontan population and in mitigating morbidity associated with advanced FALD.

## Data Availability

Data is provided within the manuscript and tables.

## Abbreviations

CHD: Congenital heart disease
EGD: Esophagogastroduodenoscopy
FALD: Fontan-associated liver disease
